# Right over left ventricular end-diastolic area relevance to predict hemodynamic intolerance of high-frequency oscillatory ventilation in patients with severe ARDS

**DOI:** 10.1186/s13613-015-0068-6

**Published:** 2015-09-17

**Authors:** Lionel Ursulet, Arnaud Roussiaux, Dominique Belcour, Cyril Ferdynus, Bernard-Alex Gauzere, David Vandroux, Julien Jabot

**Affiliations:** Medical Surgical Intensive Care Unit, Saint Denis University Hospital, Saint Denis, Reunion Island France; Methodological Support and Biostatistics Unit, Saint Denis University Hospital, Saint Denis, Reunion Island France

**Keywords:** Acute respiratory distress syndrome, High-frequency oscillatory ventilation, Hemodynamic monitoring, Acute right ventricular dysfunction, Echocardiography, Transpulmonary thermodilution

## Abstract

**Background:**

High-frequency oscillatory ventilation (HFOV) does not improve the prognosis of ARDS patients despite an improvement in oxygenation. This paradox may partly be explained by HFOV hemodynamic side-effects on right ventricular function. Our goal was to study the link between HFOV and hemodynamic effects and to test if the pre-HFOV right over left ventricular end-diastolic area (RVEDA/LVEDA) ratio, as a simple parameter of afterload-related RV dysfunction, could be used to predict HFOV hemodynamic intolerance in patients with severe ARDS.

**Methods:**

Twenty-four patients were studied just before and within 3 h of HFOV using transthoracic echocardiography and transpulmonary thermodilution.

**Results:**

Before HFOV, the mean PaO_2_/FiO_2_ ratio was 89 ± 23. The number of patients with a RVEDA/LVEDA ratio >0.6 significantly increased after HFOV [11 (46 %) vs. 17 (71 %)]. Although HFOV did not significantly decrease the arterial pressure (systolic, diastolic, mean and pulse pressure), it significantly decreased the cardiac index (CI) by 13 ± 18 % and significantly increased the RVEDA/LVEDA ratio by 14 ± 11 %. A significant correlation was observed between pre-HFOV RVEDA/LVEDA ratio and CI diminution after HFOV (*r* = 0.78; *p* < 0.0001). A RVEDA/LVEDA ratio superior to 0.6 resulted in a CI decrease >15 % during HFOV with a sensitivity of 80 % (95 % confidence interval 44–98 %) and a specificity of 79 % (confidence interval 49–95 %).

**Conclusion:**

The RVEDA/LVEDA ratio measured just before HFOV predicts the hemodynamic intolerance of this technique in patients with severe ARDS. A high ratio under CMV raises questions about the use of HFOV in such patients.

Trial registration: ClinicalTrials.gov: NCT01167621

## Background

In severe acute respiratory distress syndrome (ARDS), alternative therapies are indicated [1] when protective conventional mechanical ventilation (CMV) fails to maintain efficient gas exchange. High-frequency oscillatory ventilation (HFOV) is a non-CMV technique that could improve alveolar recruitment and achieve protective ventilation with the most severe cases of ARDS using very low tidal volume (VT) and relatively high mean airway pressure (mPaw) as a surrogate for positive end-expiratory pressure (PEEP). Previous studies have clearly shown an improvement in oxygenation with HFOV [2] and even suggest a reduction in mortality [3, 4] although the level of improvement in oxygenation is unpredictable and HFOV can indeed worsen oxygenation in some patients [5]. Alveolar recruitment—and thus improvement in gas exchange—seems to depend on the level of mPaw [6, 7] and a high mPaw (around 30 cm H_2_O) for a period of several days seems to give the best results [6]. Unfortunately, two large, multicenter, randomized controlled trials recently raised doubts about the safety of HFOV: the first [8] showed no significant effect on 30-day mortality and, troublingly, the second [9] was prematurely discontinued following a non-significant but constant increase in mortality at each interim analysis. This paradox—an improvement in oxygenation in most cases on the one hand but no mortality reduction on the other—may partly be explained by the hemodynamic side-effects of HFOV. Indeed, a retrospective study [5] involving 190 patients treated by HFOV reported a high rate of hemodynamic complications (27 %). A study involving only nine patients with high mPaw HFOV showed a decrease in cardiac index (CI), probably due to airway pressure-related preload reduction [10]. Finally, a study involving 16 patients demonstrated that HFOV could worsen right ventricular function [11]. Therefore, our goal was to monitor hemodynamic effects during the first 3 h with high mPaw HFOV using transthoracic echocardiography (TTE) and transpulmonary thermodilution (TPTD) and to assess if afterload-related RV dysfunction measured just before HFOV like the right over left ventricular end-diastolic area (RVEDA/LVEDA) ratio could predict hemodynamic intolerance during HFOV.

## Methods

### Patients

This observational prospective study was approved by the ethics committee of Bordeaux University Hospital (Comité de Protection des Personnes Sud-Ouest et Outre-Mer III, no. 2010-A00338-31).Written informed consent was obtained from each patient’s next of kin.

In our 18-bed intensive care unit (ICU), VMC therapy for ARDS was set according to a standardized protocol for maximal alveolar recruitment: protective CMV consisted of a volume-controlled mode with a VT of 6 mL kg^−1^ of predicted body weight (PBW), maximal PEEP without exceeding a plateau airway pressure of 30 cm H_2_O [12], controlled sedation for a Ramsay Score >5 [13] followed by continuous infusion of *cis*-atracurium [14] and systematic use of a heated humidifier and a closed endotracheal suction catheter [15]. All patients were hemodynamically optimized according to TPTD monitoring PiCCO2 (Pulsion Medical Systems, Munich, Germany) and/or TTE monitoring [16, 17].

In our unit, refractory ARDS was defined as follows:

- Life-threatening hypoxemia (PaO_2_/FiO_2_ <70 mmHg) at any time during the first 12 h of CMV for maximal alveolar recruitment
- When one of more of the following criteria are met after 12 h of CMV: arterial blood oxygen saturation <90 % with fraction of inspired oxygen (FiO_2_) = 1; PaO_2_/FiO_2_ ratio <120; hypercapnia >55 mmHg or pH <7.20 despite a respiratory rate of 35 min^−1^, plateau airway pressure >30 cm H_2_O with a tidal volume of 6 mL kg^−1^ of PBW and a 5 cm H_2_O minimal PEEP.

When a patient exhibited these criteria for refractory ARDS, rescue therapies were considered (i.e., HFOV, prone positioning [18] or extra-corporeal membrane oxygenation (ECMO) [19]). Patients undergoing HFOV were enrolled in the study from May 2010 to November 2012 if the following criteria were met:

- Refractory ARDS
- HFOV used as an alternative therapy to CMV
- A TPTD device used for hemodynamic monitoring
- Hemodynamic stability during the 10 min prior to HFOV (mean arterial pressure stable and between 65 and 85 mmHg, no CI variation of more than 10 % given by the PiCCO_2_ beat-to-beat pulse contour analysis, no change in norepinephrine dose).

Exclusion criteria were:

- Age <18 years
- Moribund status
- Contraindications to HFOV (head injury, pneumothorax or persistent air leak despite chest tube insertion)
- Hemodynamic instability before HFOV
- Poor echogenicity preventing appropriate echocardiographic assessment by TTE.

### HFOV

#### Initial settings and parameter adjustment

At inclusion, HFOV (3100B SensorMedics ventilator, Yorba Linda, CA, USA) was initially set as follows: FiO_2_ 1; frequency 6 Hz; bias flow 40 L min^−1^; mPaw 10 cm H_2_O above the CMV mPAw up to a maximum of 30 cm H_2_O and pressure amplitude of oscillation 80 %. During the 3-h protocol, mPaw and bias flow were not modified; other parameters were adjusted as follows:

- FiO_2_ adjusted to obtain a PaO_2_ of 60–85 mmHg
- Frequency and pressure amplitude of oscillation adjusted to obtain a pH >7.25 and a PCO_2_ <55 mmHg

#### HFOV failure criteria

HFOV was defined as a failure if at least one of the following occurred:

- Oxygen saturation fell below 90 % despite a FiO_2_ 1
- PaO_2_/FiO_2_ ratio <70
- Hypercapnia >55 mmHg and/or pH <7.20 despite a frequency of 3.5 Hz and pressure amplitude of oscillation of 100 %
- Occurrence of a new pneumothorax.

We defined HFOV-associated hemodynamic failure as major hemodynamic instability linked to HFOV: significant arterial hypotension (systolic arterial pressure under 90 mmHg or a decrease >30 % of initial systolic pressure) and/or CI decrease >30 %.

When HFOV failure was ascertained, CMV was removed leading to the termination of the study and an alternative method of oxygenation (prone positioning or ECMO) was initiated. If systolic arterial pressure was under 90 mmHg, the norepinephrine dose was increased during the HFOV–CMV transfer process.

### Hemodynamic measurements

The study began at patient inclusion, i.e., under CMV during a 10-min period before initiation of HFOV, and ended 3 h later. The different stages of the study were:

- Just before initiation of HFOV (CMVpre)
- Connection to HFOV (HFOV connection)
- After 1 h of HFOV (H_1 HFOV_)
- After 3 h of HFOV (H_3 HFOV_)

In each sequence, heart rate, arterial pressure, catecholamine and sedation dose rates and TTE and TPTD data were recorded.

#### TTE measurements

TTE were performed by a single experienced echocardiographer. Images were acquired using an EnVisor Philips HD 11XE (Philips Medical System, Andover, MA, USA) scanner and a 3 MHz transducer. Two-dimensional (2D) imaging examinations were performed in the standard apical four- and two-chamber views (4C- and 2C-views). Tissue harmonic imaging was used to enhance 2D image quality. Left ventricular ejection fraction (LVEF) was measured either by the biplane or monoplane Simpson method [20]. The velocity–time integral in the left ventricular outflow tract (LVOT VTI) was measured using pulsed wave Doppler in the apical five-chamber view. LVEDA and RVEDA were measured in the 4C-view. Echocardiographic patterns of acute cor pulmonale (ACP) associating RVEDA/LVEDA ratio >0.6 and systolic septal dyskinesia on a short-axis view were looked for [21]. LV filling parameters were assessed in the 4C-view and using pulsed wave and tissue Doppler imaging in accordance with the current standards [22, 23]. All TTE studies were recorded over three consecutive cardiac cycles independently of the respiratory cycle and averaged. In patients with non-sinus rhythm, measurements were collected over 5–7 heartbeats.

#### TPTD measurements

Injection of 15 mL of cold saline through the central venous line was performed in triplicate, and the values of the different TDTP parameters [CI, stroke volume index (SVI), global end-diastolic index (GEDI) and cardiac function index (CFI)] were averaged [24].

### Respiratory measurements

For each sequence, ventilator settings, respiratory system mechanical parameters, arterial blood gas analysis, extravascular lung water index (ELWI) and pulmonary vascular permeability water index (PVPI) obtained by TPTD were collected [25, 26].

### Statistical analysis

Qualitative variables were described in frequencies and proportions. Quantitative variables were described in means and standard deviations. Evolution of hemodynamic and respiratory levels during the study was assessed using a linear mixed model adjusted for time. A first-order autoregressive variance–covariance matrix was specified, to account for the correlated repeated time data. All multiple comparisons were performed using the Scheffe adjustment. The receiver operating characteristics curve was constructed to assess the ability of an RVEDA/LVEDA ratio at inclusion to predict a decrease in CI >15 % with HFOV. Spearman rank correlation analysis was used to assess the relationship between RVEDA/LVEDA ratio at inclusion and changes in CI on HFOV. Statistical analysis was performed using the SAS 9.2 software (SAS Institute, Cary, NC, USA). All hypotheses were tested at the 2-tailed 0.05 significance level.

## Results

Twenty-four patients (7 women and 17 men) were included in the study. None of them had a history of chronic respiratory failure. The mean time between ICU-CMV and HFOV was 9 ± 4 h. Twenty-three patients were still under HFOV at H_1_ (one patient was withdrawn for hemodynamic failure), and 19 at H_3_ (two patients were withdrawn for respiratory failure and two more for hemodynamic failure).

On admission, the Simplified Acute Physiology Score II (SAPS II) and the Sequential Organ Failure Assessment (SOFA) were, respectively, 60 ± 17 and 12 ± 3. Mortality at D28 was 46 %. Causes of ARDS were pulmonary for 75 % of patients (*n* = 18) (thirteen cases of infectious pneumonia, four aspiration pneumonia and one drowning accident), and extra-pulmonary for 25 % of patients (*n* = 6). At baseline, 21 patients (82 %) were receiving norepinephrine and three dobutamine. The respiratory variables during CMV at baseline are summarized in Table 1.

**Table 1:** Respiratory variables at baseline

Ventilator settings	
VT (mL kg^−1^ PBW)	5.8 ± 0.6
Respiratory rate (cycles min^−1^)	29 ± 3
PEEP (cm H_2_O)	11 ± 3
FiO_2_ (%)	97 ± 9
Respiratory-system mechanics	
Plateau airway pressure (cm H_2_O)	29 ± 2
mPaw (cm H_2_O)	19 ± 3
Respiratory system compliance (mL cm H_2_O^−1^)	22 ± 9
Results of ABG measurements	
pH	7.24 ± 0.14
P/F ratio	89 ± 23
PaO_2_ (mmHg)	86 ± 22
PaCO_2_ (mmHg)	53 ± 15
Bicarbonate (mmol L^−1^)	24 ± 5
Base excess (mmol L^−1^)	−6 ± 6
TDTP respiratory parameters	
ELWI (mL kg^−1^ PBW)	19 ± 7
PVPI	5.1 ± 1.7

Results are given as mean ± SD

*ABG* arterial blood gas, *TPTD* transpulmonary thermodilution, *VT* tidal-volume, *PBW* predicted body weight, PEEP positive end-expiratory pressure, *FiO*_*2*_ fraction of inspired oxygen, *mPaw* mean airway pressure, *P/F* ratio of arterial oxygen concentration to the fraction of inspired oxygen, *PaCO*_*2*_ partial pressure of arterial carbon dioxide, *PaO*_*2*_ partial pressure of arterial oxygen, *ELWI* extravascular lung water index, *PVPI* pulmonary vascular permeability index

### Hemodynamic parameters

#### Rate and cause of hemodynamic failure

Hemodynamic failure was reported in three patients (12 %), occurring within the first 90 min after HFOV. These three patients had an ACP echocardiographic pattern not only during CMV but also after HFOV.

#### Hemodynamic effects of HFOV

**Fig. 1:**
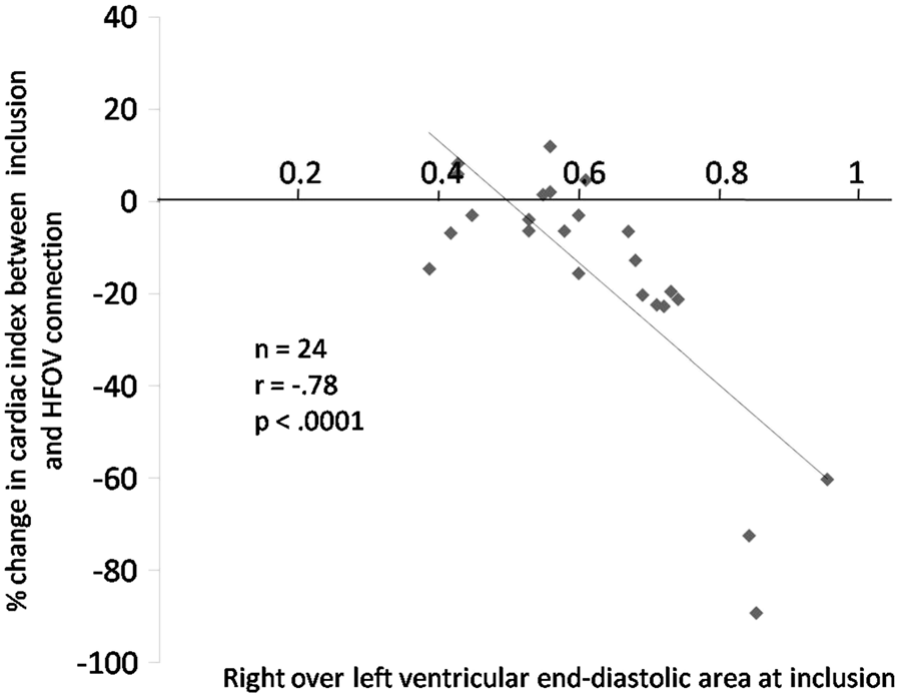
Inverse correlation between the right over left ventricular end-diastolic area at inclusion and changes in cardiac index during HFOV. *Line* linear regression line

**Fig. 2:**
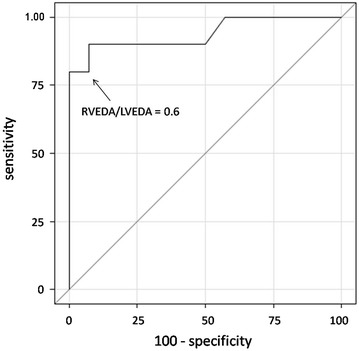
Receiver operating characteristic curve showing the ability of the right over left ventricular end-diastolic area at inclusion to detect a cardiac index decrease ≥15 % during HFOV. *RVEDA* right ventricular end diastolic area, *LVEDA* left ventricular end diastolic area

As shown in Table 2, although HFOV did not significantly decrease arterial pressure (systolic, diastolic, mean and pulse pressure), it significantly decreased CI by 13 ± 11 % from 3.7 ± 1.1 L min^−1^ m^−2^ at baseline and LVOT VTI by 13 ± 12 % from 17 ± 5 cm at baseline. SVI, GEDI, CFI, LVEF and E/A ratio also significantly decreased, whereas the RVEDA/LVEDA ratio increased by 14 ± 11 %, from 0.61 ± 0.15 at baseline. The number of patients with an RVEDA/LVEDA ratio >0.6 and with an ACP echocardiographic pattern significantly increased during HFOV [11 (46 %) vs. 17 (71 %) and 5 (21 %) vs. 11 (46 %), respectively]. For each patient, RVEDA/LVEDA ratio on inclusion was compared with the percentage change in TDTP CI between inclusion and HFOV. When considering these 24 pairs of measurements, a significant inverse correlation was observed (*r* = −0.78; *p* < 0.0001) (Fig. 1). An RVEDA/LVEDA ratio superior to 0.6 predicted a decrease in CI >15 % during HFOV with a sensitivity of 80 % (95 % confidence interval 44–98 %) and a specificity of 79 % (confidence interval 49–95 %) (Fig. 2).

**Table 2:** Evolution of the hemodynamic characteristics during the study

	CMV_pre_	HFOV connection	H_1 HFOV_	H_3 HFOV_
Concerned patients number	24	24	23	19
Heart rate (beats/min)	102 ± 22	102 ± 23	102 ± 23	102 ± 24
Systolic arterial pressure (mmHg)	120 ± 18	119 ± 23	119 ± 19	116 ± 17
Diastolic arterial pressure (mmHg)	62 ± 11	63 ± 12	65 ± 11	62 ± 11
Mean arterial pressure (mmHg)	81 ± 11	81 ± 13	80 ± 14	80 ± 12
Pulse pressure (mmHg)	57 ± 13	56 ± 17	54 ± 17	54 ± 13
Cardiac index (L min^−1^ m^−2^) (TPTD)	3.7 ± 1.1^b,c,d^	3.3 ± 1.3^a^	3.3 ± 1.2^a^	3.1 ± 1.1^a^
SVI (mL min^−1^ m^−2^) (TPTD)	36 ± 11^b,c,d^	33 ± 14^a^	33 ± 14^a^	32 ± 14^a^
GEDI (mL min^−1^ m^−2^) (TPTD)	680 ± 140^b,c,d^	634 ± 134^a^	646 ± 126^a^	625 ± 112^a^
CFI (min^−1^) (TPTD)	5.5 ± 1.8^b,c,d^	5.2 ± 1.9^a^	5.0 ± 1.8^a^	5.0 ± 1.5^a^
LVEF (%) (TTE)	53 ± 16^b,c,d^	50 ± 17^a^	49 ± 15^a^	49 ± 13^a^
RVEDA/LVEDA ratio (TTE)	0.61 ± 0.15^b,c,d^	0.70 ± 0.18^a^	0.72 ± 0.18^a^	0.67 ± 0.14^a^
RVEDA/LVEDA ratio >0.6 [*n* (%)]	11 (46)^b,c^	17 (71)^a,d^	17 (74)^a,d^	10 (53)^b,c^
ACP echocardiographic pattern [*n* (%)]	5 (21)^b,c^	11 (46)^a,d^	10 (43)^a,d^	6 (32)^b,c^
LVOT VTI (cm) (TTE)	17 ± 5^b,c,d^	14 ± 5^a^	14 ± 5^a^	14 ± 5^a^
E-wave (cm s^−1^) (TTE)	90 ± 23^c^	86 ± 20^c^	80 ± 21^a,b,d^	91 ± 22^c^
A-wave (cm s^−1^) (TTE)	57 ± 16	58 ± 12	54 ± 12	57 ± 15
E′ (cm s^−1^) (TTE)	14 ± 5	14 ± 5	14 ± 4	15 ± 5
DTE (ms) (TTE)	207 ± 51	204 ± 44	213 ± 38	205 ± 46
E/A (TTE)	1.8 ± 0.5^b,c^	1.6 ± 0.6^a^	1.6 ± 0.5^a^	1.8 ± 0.7^b,c^
E/E′ (TTE)	8.3 ± 2.9	8.4 ± 2.9	7.4 ± 3.6	8.0 ± 3.0
Norepinephrine (μg kg^−1^ min^−1^)	0.59 ± 0.78	0.59 ± 0.78	0.58 ± 0.78	0.53 ± 0.69

Results are given as mean ± SD

*CMV* conventional mechanical ventilation, *HFOV* high frequency oscillation ventilation, *TPTD* transpulmonary thermodilution, *SVI* stroke volume index, *GEDI* global end diastolic index, *CFI* cardiac function index, *LVEF* left ventricular ejection fraction, *TTE* transthoracic echocardiography, *LVEDA* left ventricular end diastolic area, *RVEDA* right ventricular end diastolic area, *LVOT VTI* velocity–time integral in the left ventricular outflow tract, *DTE* E-wave deceleration time

^a^ *p* < 0.05 for all data as compared to CMVpre

^b^ *p* < 0.05 for all data as compared to HFOV Connection

^c^ *p* < 0.05 for all data as compared to H_1__HFOV_

^d^ *p* < 0.05 for all data as compared to H_3__HFOV_

#### Hemodynamic changes during the first 3 h of HFOV

There was no hemodynamic change during the first 3 h with the exception of E-wave, E/A ratio, RVEDA/LVEDA ratio and numbers of ACP echocardiographic patterns (Table 2).

#### Therapeutic interventions by the attending physicians

During the 3-h study, no volume expansion was administered to patients and there was no significant change in doses of catecholamines, sedative drugs or cisatracurium. Therapeutic interventions were carried out only on the three patients with HFOV-associated hemodynamic failure.

### Respiratory parameters

Respiratory parameter changes are summarized in Table 3. The comparison of PaO_2_/FiO_2_ ratios revealed a significant increase of 90 % or more between the HFOV sequences and CMVpre sequence.

**Table 3:** Evolution of the respiratory and gazometric parameters during the study

	CMV_pre_	HFOV connection	H_1 HFOV_	H_3 HFOV_
Concerned patients number	24	24	23	19
mPaw (cm H_2_O)	19 ± 3^b,c,d^	29 ± 1^a^	28 ± 1^a^	29 ± 1^a^
Frequency (Hz)	NA	6.0 ± 0.0^d^	6.0 ± 0.0^d^	5.2 ± 1.3^b,c^
Amplitude (cm H_2_O)	NA	88 ± 13	86 ± 12	85 ± 12
Pressure amplitude of oscillation (%)	NA	80 ± 0	79 ± 4	79 ± 5
pH	7.24 ± 0.14^d^	NA	7.25 ± 0.16	7.30 ± 0.17^a^
PaCO_2_ (mmHg)	53 ± 15^d^	NA	49 ± 19^a^	47 ± 15^a^
Bicarbonate (mmol L^−1^)	24 ± 5	NA	23 ± 6	24 ± 4
Base excess (mmol L^−1^)	−6 ± 6^d^	NA	−6 ± 7^d^	−4 ± 5^a,c^
P/F ratio	89 ± 23^c,d^	NA	171 ± 106^a^	177 ± 96^a^
OI	26 ± 8^d^	NA	26 ± 17^d^	23 ± 15^a,c^
ELWI (mL/kg PBW)	19 ± 7	19 ± 7	19 ± 7	17 ± 6
PVPI	5.1 ± 1.7	5.2 ± 1.8	5.1 ± 1.5	5.1 ± 1.7

Results are given as mean ± SD

*CMV* conventional mechanical ventilation, *HFOV* high frequency oscillation ventilation, *mPaw* mean airway pressure, *FiO*_*2*_ fraction of inspired oxygen, *P*/*F* ratio of arterial oxygen concentration to the fraction of inspired oxygen, *OI* oxygenation index calculated as (mean airway pressure × FiO_2_)/PaO_2_, *ELWI* extravascular lung water index, *PBW* predicted body weight, *PVPI* pulmonary vascular permeability index

^a^ *p* < 0.05 for all data as compared to CMV_pre_

^b^ *p* < 0.05 for all data as compared to HFOV connection

^c^ *p* < 0.05 for all data as compared to H_1__HFOV_

^d^ *p* < 0.05 for all data as compared to H_3__HFOV_

At H_1 HFOV_, the PaO_2_/FiO_2_ ratio increased from the baseline value by more than 100 % for 39 % of patients and by more than 30 % for 61 % of patients.

At H_3 HFOV_, the PaO_2_/FiO_2_ ratio increased from the baseline value by more than 100 % for 47 % of patients and by more than 30 % for 74 % of patients.

During the study, there was no significant difference between the mean PaO_2_/FiO_2_ ratios of the 18 patients with pulmonary ARDS and the 6 patients with extra-pulmonary ARDS (91 ± 22 vs. 83 ± 27 at inclusion, 180 ± 104 vs. 147 ± 119 at H_1_ and 186 ± 81 vs. 158 ± 131 at H_3_, respectively).

## Discussion

To our knowledge, this study is the largest that focuses specifically on hemodynamic changes during HFOV. It confirms the significant CI decrease linked to HFOV. It also suggests that the initial pre-HFOV RVEDA/LVEDA ratio can predict the hemodynamic intolerance induced by HFOV.

Although right heart dysfunction is a commonly reported side-effect of ARDS protective ventilation [27], only two prospective studies have specifically focused on the hemodynamic effects of HFOV, with both transesophageal echocardiography (TEE) and right heart catheterization monitoring. David et al. reported a clinically significant decrease in CI and SVI thought to be related to a preload decrease [10]. Guervilly et al. described a right ventricular dysfunction in HFOV proportional to the mPaw setting level [11].

Our results are in accordance with those two studies, since HFOV led to a 13 % decrease in CI and to a 14 % increase of RVEDA/LVEDA. The proportion of patients with a ratio above 0.6 (46 %) is comparable with that reported by Guervilly et al. (56 %) [11].

The initial pre-HFOV RVEDA/LVEDA ratio is a good predictive factor of HFOV hemodynamic intolerance, as shown by the strong correlation of 78 % (*r*^2^ = 0.61) between the pre-HFOV ratio and the CI decrease induced by HFOV and the sensitivity (80 %) and specificity (79 %) values found to predict a decrease in 15 % CI by a CMV pre-HFOV RVEDA/LVEDA superior to 0.6. Such results underline the value of performing an echocardiography before HFOV to assess the RVEDA/LVEDA ratio and the risk of right ventricular dysfunction. In our study, the exact pathophysiology of right ventricular dysfunction cannot be explained given the lack of pulmonary vascular resistance monitoring.

Although our definition of refractory ARDS is neither published nor consensual, these patients had severe ARDS after 12 h of CMV maximum alveolar recruitment with a mean PaO_2_/FiO_2_ ratio of 89 ± 23. This new practice of delaying ARDS severity ranking is of key importance as it precludes inclusion of initially severe ARDS patients showing rapid improvement with CMV once set to maximal alveolar recruitment. Although our study was designed before the new Berlin definition for ARDS [28], its HFOV utilization is very close to that proposed by experts [29]. The early and very brief use of HFOV for 3 h for severe ARDS patients improved oxygenation in 66 % of cases. These data are consistent with a recent retrospective study [30] in which the early response to HFOV (an improvement of more than 38 % in the PaO_2_/FiO_2_ ratio) was identified as a predictor for survival at day 30. Therefore, HFOV could be used in the future as a recruitment technique, possibly applied sequentially as for prone positioning. This new approach is currently being studied in our ICU.

It must be said that the two recently published large randomized controlled trials, OSCILLATE [9] and OSCAR [8] comparing HFOV with a conventional lung-protective ventilation, casted doubt over HFOV, suggesting no benefit or even a worse outcome on adults with HFOV with early moderate to severe ARDS. But despite strong compliance with current recommendations of protective ventilation from the ARDS network [31] and prompt initiation of HFOV in these two studies, randomization was performed on patients with moderate to severe ARDS (PaO_2_/FiO_2_ <200), not severe ARDS patients only. Furthermore, the randomization was conducted regardless of early clinical evolution under CMV and the use of muscle relaxants was practitioner dependant. In the OSCILLATE study [9], the use of very high mPaw, without ruling out risk of potential hemodynamic failure, may have led to an excess mortality in the HFOV arm of this study. These two studies have given rise to three recent meta-analyses including 6 RCTs for 1608 patients [32], 5 RCTs for 1580 patients [33] and 7 RCTs for 1759 patients [34] without confirming better survival or higher mortality with HFOV.

Our study has several limitations. Its observational design with non-consecutive patients could engender a selection bias. The study population is small given the monocentric screening and the stringent inclusion criteria. Even if only a single experienced operator performed TTE, a post hoc analysis by an independent expert could have confirmed the hemodynamic data obtained by blinded practitioners. Similarly, data were obtained by TTE and not TEE, which, in the context of HFOV, could cast doubts on the accuracy of the results. Yet, TTE-obtained data are consistent with those found by TPTD (e.g., LVOT VTI and TPTD CI both decreased by 13 % on HFOV) and comparable to those obtained by Guervilly et al. with TEE [11]. Another limitation of the study is the lack of assessment of a potential preload decrease during HFOV. The only way to test this would have been a comparison of the passive leg raising results at inclusion and during HFOV since pulse pressure variation, stroke volume variation, vena cava variations and tele-expiratory occlusion test cannot be used with HFOV. Unfortunately, this test is very difficult to implement with HFOV and would have slowed and complicated the protocol. Lastly, it is unfortunate that we could not compare TTE data with those obtained by a pulmonary artery catheter (PAC) to better assess right ventricular function. However, our study was strictly observational and we routinely use TPTD and not PAC for monitoring patients with severe ARDS. This enables us to better assess the risk/benefit ratio of volume expansion based on ELWI and PVPI rather than on pulmonary capillary pressure [35, 36]. In this context, we considered it unethical to insert a PAC in addition to a TPTD device only for the purpose of the study.

## Conclusion

This study suggests that the RVEDA/LVEDA ratio measured just before HFOV is a predictor of the hemodynamic intolerance of this technique in patients with severe ARDS. A high value of this ratio observed under CMV should question the use of HFOV in such patients.

## Abbreviations

ARDS: acute respiratory distress syndrome
CMV: conventional mechanical ventilation
HFOV: high-frequency oscillatory ventilation
MPaw: mean airway pressure
CI: cardiac index
TTE: transthoracic echocardiography
TPTD: transpulmonary thermodilution
RVEDA/LVEDA: right over left ventricular end-diastolic area
ICU: intensive care unit
PBW: predicted body weight
PEEP: positive end-expiratory pressure
2D: two dimensional
2C-view: two-chamber view
4C-view: four-chamber view
LVEF: left ventricular ejection fraction
LVOT VTI: velocity–time integral in the left ventricular outflow tract
ACP: acute cor pulmonale
ELWI: extravascular lung water index
PVPI: pulmonary vascular permeability water index
SVI: stroke volume index
GEDI: global end-diastolic index
CFI: cardiac function index
SAPS II: Simplified Acute Physiology Score II
SOFA: sequential organ failure assessment
PAC: pulmonary artery catheter

## References

[1] Ferguson ND, Fan E, Camporota L, Antonelli M, Anzueto A, Beale R (2012). The Berlin definition of ARDS: an expanded rationale, justification, and supplementary material. Intensive Care Med.

[2] Niwa T, Hasegawa R, Ryuge M, Kawase M, Kondoh Y, Taniguchi H (2011). Benefits and risks associated with the R100 high frequency oscillatory ventilator for patients with severe hypoxaemic respiratory failure. Anaesth Intensive Care.

[3] Mentzelopoulos SD, Malachias S, Zintzaras E, Kokkoris S, Zakynthinos E, Makris D (2012). Intermittent recruitment with high-frequency oscillation/tracheal gas insufflation in acute respiratory distress syndrome. Eur Respir J.

[4] Sud S, Sud M, Friedrich JO, Meade MO, Ferguson ND, Wunsch H (2010). High frequency oscillation in patients with acute lung injury and acute respiratory distress syndrome (ARDS): systematic review and meta-analysis. BMJ.

[5] Adhikari NKJ, Bashir A, Lamontagne F, Mehta S, Ferguson ND, Zhou Q (2011). High-frequency oscillation in adults: a utilization review. Crit Care Med.

[6] Derdak S, Mehta S, Stewart TE, Smith T, Rogers M, Buchman TG (2002). High-frequency oscillatory ventilation for acute respiratory distress syndrome in adults: a randomized, controlled trial. Am J Respir Crit Care Med.

[7] Papazian L, Gainnier M, Marin V, Donati S, Arnal J-M, Demory D (2005). Comparison of prone positioning and high-frequency oscillatory ventilation in patients with acute respiratory distress syndrome. Crit Care Med.

[8] Young D, Lamb SE, Shah S, MacKenzie I, Tunnicliffe W, Lall R (2013). High-frequency oscillation for acute respiratory distress syndrome. N Engl J Med.

[9] Ferguson ND, Cook DJ, Guyatt GH, Mehta S, Hand L, Austin P (2013). High-frequency oscillation in early acute respiratory distress syndrome. N Engl J Med.

[10] David M, von Bardeleben RS, Weiler N, Markstaller K, Scholz A, Karmrodt J (2004). Cardiac function and haemodynamics during transition to high-frequency oscillatory ventilation. Eur J Anaesthesiol.

[11] Guervilly C, Forel J-M, Hraiech S, Demory D, Allardet-Servent J, Adda M (2012). Right ventricular function during high-frequency oscillatory ventilation in adults with acute respiratory distress syndrome. Crit Care Med.

[12] Mercat A, Richard J-CM, Vielle B, Jaber S, Osman D, Diehl J-L (2008). Positive end-expiratory pressure setting in adults with acute lung injury and acute respiratory distress syndrome: a randomized controlled trial. JAMA.

[13] Van Dishoeck A-M, van der Hooft T, Simoons ML, van der Ent M, Scholte OP, Reimer WJM (2009). Reliable assessment of sedation level in routine clinical practice by adding an instruction to the Ramsay Scale. Eur J Cardiovasc Nurs.

[14] Papazian L, Forel J-M, Gacouin A, Penot-Ragon C, Perrin G, Loundou A (2010). Neuromuscular blockers in early acute respiratory distress syndrome. N Engl J Med.

[15] Morán I, Bellapart J, Vari A, Mancebo J (2006). Heat and moisture exchangers and heated humidifiers in acute lung injury/acute respiratory distress syndrome patients. Effects on respiratory mechanics and gas exchange. Intensive Care Med.

[16] Allyn J, Allou N, Dib M, Tashk P, Desmard M, Dufour G (2013). Echocardiography to predict tolerance to negative fluid balance in acute respiratory distress syndrome/acute lung injury. J Crit Care.

[17] Isakow W, Schuster DP (2006). Extravascular lung water measurements and hemodynamic monitoring in the critically ill: bedside alternatives to the pulmonary artery catheter. Am J Physiol Lung Cell Mol Physiol.

[18] Guérin C, Reignier J, Richard J-C, Beuret P, Gacouin A, Boulain T (2013). Prone positioning in severe acute respiratory distress syndrome. N Engl J Med.

[19] Richard C, Argaud L, Blet A, Boulain T, Contentin L, Dechartres A (2014). Extracorporeal life support for patients with acute respiratory distress syndrome: report of a Consensus Conference. Ann Intensive Care.

[20] Lang RM, Bierig M, Devereux RB, Flachskampf FA, Foster E, Pellikka PA (2005). Recommendations for chamber quantification: a report from the American Society of Echocardiography’s Guidelines and Standards Committee and the Chamber Quantification Writing Group, developed in conjunction with the European Association of Echocardiograph. J Am Soc Echocardiogr.

[21] Vieillard-Baron A, Schmitt JM, Augarde R, Fellahi JL, Prin S, Page B (2001). Acute cor pulmonale in acute respiratory distress syndrome submitted to protective ventilation: incidence, clinical implications, and prognosis. Crit Care Med.

[22] Nagueh SF, Appleton CP, Gillebert TC, Marino PN, Oh JK, Smiseth OA (2009). Recommendations for the evaluation of left ventricular diastolic function by echocardiography. Eur J Echocardiogr.

[23] Paulus WJ, Tschöpe C, Sanderson JE, Rusconi C, Flachskampf FA, Rademakers FE (2007). How to diagnose diastolic heart failure: a consensus statement on the diagnosis of heart failure with normal left ventricular ejection fraction by the Heart Failure and Echocardiography Associations of the European Society of Cardiology. Eur Heart J.

[24] Sakka SG, Reuter DA, Perel A (2012). The transpulmonary thermodilution technique. J Clin Monit Comput.

[25] Sakka SG, Rühl CC, Pfeiffer UJ, Beale R, McLuckie A, Reinhart K (2000). Assessment of cardiac preload and extravascular lung water by single transpulmonary thermodilution. Intensive Care Med.

[26] Monnet X, Anguel N, Osman D, Hamzaoui O, Richard C, Teboul J-L (2007). Assessing pulmonary permeability by transpulmonary thermodilution allows differentiation of hydrostatic pulmonary edema from ALI/ARDS. Intensive Care Med.

[27] Vieillard-Baron A, Price LC, Matthay MA (2013). Acute cor pulmonale in ARDS. Intensive Care Med.

[28] Ranieri VM, Rubenfeld GD, Thompson BT, Ferguson ND, Caldwell E, Fan E (2012). Acute respiratory distress syndrome: the Berlin definition. JAMA.

[29] Ferguson ND, Fan E, Camporota L, Antonelli M, Anzueto A, Beale R (2012). The Berlin definition of ARDS: an expanded rationale, justification, and supplementary material. Intensive Care Med.

[30] Camporota L, Sherry T, Smith J, Lei K, McLuckie A, Beale R (2013). Physiological predictors of survival during high-frequency oscillatory ventilation in adults with acute respiratory distress syndrome. Crit Care.

[31] Ventilation with lower tidal volumes as compared with traditional tidal volumes for acute lung injury and the acute respiratory distress syndrome. The acute respiratory distress syndrome network. N Engl J Med. 2000;342:1301–1308. doi:10.1056/NEJM200005043421801. 10.1056/NEJM200005043421801 10793162

[32] Gu X-L, Wu G-N, Yao Y-W, Shi D-H, Song Y (2014). In adult acute respiratory distress syndrome patients, is high-frequency oscillatory ventilation more effective and safer than conventional protective ventilation? A meta-analysis of randomized controlled trials. Crit Care.

[33] Huang C-T, Lin H-H, Ruan S-Y, Lee M-S, Tsai Y-J, Yu C-J (2014). Efficacy and adverse events of high frequency oscillatory ventilation in adult patients with acute respiratory distress syndrome: a meta-analysis. Crit Care.

[34] Maitra S, Bhattacharjee S, Khanna P, Baidya DK (2014). High-frequency ventilation does not provide mortality benefit in comparison with conventional lung-protective ventilation in acute respiratory distress syndrome: a meta-analysis of the randomized controlled trials. Anesthesiology.

[35] Kushimoto S, Taira Y, Kitazawa Y, Okuchi K, Sakamoto T, Ishikura H (2012). The clinical usefulness of extravascular lung water and pulmonary vascular permeability index to diagnose and characterize pulmonary edema: a prospective multicenter study on the quantitative differential diagnostic definition for acute lung injury/acute. Crit Care.

[36] Tagami T, Nakamura T, Kushimoto S, Tosa R, Watanabe A, Kaneko T (2014). Early-phase changes of extravascular lung water index as a prognostic indicator in acute respiratory distress syndrome patients. Ann Intensive Care.

